# Lab-on-chip technology for chronic disease diagnosis

**DOI:** 10.1038/s41746-017-0014-0

**Published:** 2018-04-11

**Authors:** Jiandong Wu, Meili Dong, Claudio Rigatto, Yong Liu, Francis Lin

**Affiliations:** 1 Department of Physics and Astronomy, University of Manitoba, Winnipeg, MB Canada; 2 Institute of Applied Technology, Hefei Institutes of Physical Science, Chinese Academy of Sciences, Hefei, Anhui China; 3 Seven Oaks General Hospital, Winnipeg, MB Canada

**Keywords:** Diagnostic markers, Biomedical engineering, Diagnosis

## Abstract

Various types of chronic diseases (CD) are the leading causes of disability and death worldwide. While those diseases are chronic in nature, accurate and timely clinical decision making is critically required. Current diagnosis procedures are often lengthy and costly, which present a major bottleneck for effective CD healthcare. Rapid, reliable and low-cost diagnostic tools at point-of-care (PoC) are therefore on high demand. Owing to miniaturization, lab-on-chip (LoC) technology has high potential to enable improved biomedical applications in terms of low-cost, high-throughput, ease-of-operation and analysis. In this direction, research toward developing new LoC-based PoC systems for CD diagnosis is fast growing into an emerging area. Some studies in this area began to incorporate digital and mobile technologies. Here we review the recent developments of this area with the focus on chronic respiratory diseases (CRD), diabetes, and chronic kidney diseases (CKD). We conclude by discussing the challenges, opportunities and future perspectives of this field.

## Introduction

Chronic diseases (CD), which are characterized by their prolonged disease period with persistent symptoms, are one of the leading causes for death worldwide,^1^ and account for 70% of all healthcare expenditures.^2^ CD present a major health threat to both the aging population and the youth; and CD have a higher chance of leading to death for patients in source-limited regions according to statistics from the World Health Organization (WHO).^3^ Through the course of CD progression, patients often suffer from various acute health problems and complications, which results in lower life quality.^4^ Some of the most prominent CD are chronic respiratory diseases (CRD), diabetes, and chronic kidney diseases (CKD).^5^ Timely CD diagnosis, accurate risk prediction and frequent disease monitoring combined with effective early treatment have the promise to improve the management of CD and even prevent their progression.^6^ However, the attractive concept of early CD diagnosis is often technically challenged by the lengthy and expensive diagnostic procedures in the current clinical practice. Furthermore, the delay in obtaining CD diagnosis results presents a major limiting factor for optimizing healthcare resource allocation and coordination, which leads to significantly increased economic burden.^7^ In this context, rapid point-of-care (PoC) diagnosis is the key for improving the management of chronic health problems.

CD diagnosis, prognosis and monitoring require effective measurements of biomarkers.^8,9^ Some commonly used conventional methods for disease biomarker detection include enzyme-linked immunosorbent assay (ELISA),^10^ mass spectrometry (MS),^11^ chromatography,^12^ gel electrophoresis,^13^ and polymerase chain reaction (PCR).^14,15^ ELISA quantifies the concentration of the target protein biomarker by measuring the reporter signal with different optical substrates.^16^ Compared with other immunoassays, ELISA has the advantages in sensitivity and specificity, making it the gold standard for clinical CD diagnosis of protein biomarkers.^17,18^ MS is another important method for characterization and sequencing of protein biomarkers from minute amount of sample; and it can be combined with other techniques to test complex mixtures of protein samples.^19^ PCR, which was first developed in 1983 by Kary Mullis,^20^ has become the current standard of genetic diagnosis method for diseases.^21^ PCR-based diagnostic test has high sensitivity and high yielding of signal amplification for detecting the target genes. Various diagnosis methods such as flow cytometry, immunostaining, and cell culture have been employed for evaluating cell and tissue samples.^22–24^ More recently, new cell functional test-based approaches were developed for disease assessment.^25^ In the current clinical practice, most diagnostic tests of CD biomarkers are done in centralized labs. The major limitation of lab-based test is the long turn-around time. For example, in the scenario of CKD diagnosis, timely blood test is usually required to assess renal failure status. For lab-based test, the patient must travel to the hospital to have the blood drawn. The specimen is then sent for lab test, after which the result is provided to the physician. In this scenario, patients typically cannot obtain the test result until the next day. Some other common limitations of the conventional methods are the high-cost, complicated assay procedures, low test speed, and the requirement of specialized skills. To overcome these limitations, PoC devices have been increasingly developed. For example, the PoC blood glucose meters have been widely used for diabetes diagnosis and management.^26^ Ideally, the PoC tests should meet the ASSURED (affordable, sensitive, specific, user-friendly, rapid and robust, equipment-free and delivered) standard published by the WHO. However, so far there is no PoC test that has fully met the ASSURED criteria for biomarkers measurement. The main drawback of the current PoC methods is the reduced accuracy and still relatively high cost for resource-limited regions.^26^ Thus, researchers are motivated to develop new CD diagnosis tools suitable for rapid, reliable and cost-efficient PoC test.^27^

**Fig. 1:**
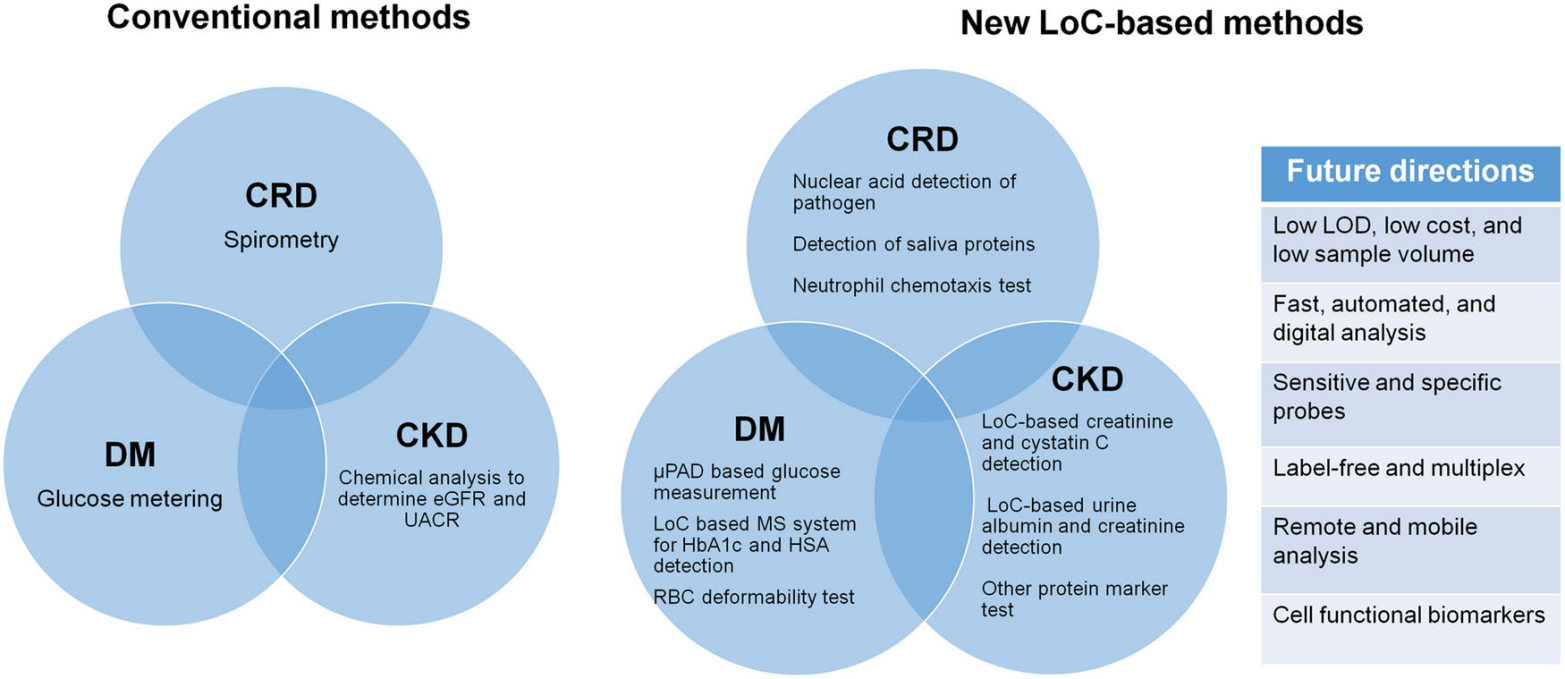
Summary of conventional and LoC-based diagnostic methods for CRD, diabetes and CKD, and future directions

Lab-on-chip (LoC) technology enables advanced biological sample processing, manipulation and analysis in miniaturized fluidic devices.^28,29^ The major advantages of LoC technology compared with conventional analytical detection methods include: (1) lower fluid volume consumption, which permits lower reagent cost and less sample volume for the experiments; (2) faster analysis time due to better-controlled fluidic transport and high surface-to-volume ratio; (3) more compact and higher experimental throughput owing to integration. Most of the LoC technologies take advantage of microfluidics, which provides the unique ability to manipulate minute amounts of fluids in micrometer scale channels. Consistently, LoC has become the most important application area of microfluidics. It is worth pointing out that LoC-based applications can also be achieved by other technologies, such as nanofluidics and millifluidics. Over the past nearly two decades, LoC technology has emerged as an important research field in biomedical engineering.^30–32^ More recently, LoC technology is fast developing toward medical applications, such as disease diagnosis,^33^ controlled drug delivery,^34^ and organ-on-chip models.^35^The combined attractive features of LoC technology in low sample and reagent consumption, high-throughput, low-cost, integration and portability make it suitable for CD diagnosis at PoC.^36–39^ Indeed, we observed significant development of LoC-based diagnostic applications for various CD over recent years. Giving the broad field of CD, in this review, we focus on LoC-based CD diagnosis for CRD, diabetes and CKD that involve the use of microfluidic devices (Fig. 1 and Table 1). Furthermore, we discuss the challenges, opportunities and future perspectives of this emerging field.

**Table 1:** Examples of LoC-based diagnostic applications for CRD, diabetes and CKD over recent years

Disease	Marker	Technique	LOD	Detector	Assay time	Sample volume	Ref
CRD	VEGF	Bead-based immunoassay	14 pg/ml	SDReader (CCD camera)	70 min	10 μl	54
	IP-10		30 pg/ml				
	IL-8		6 pg/ml				
	EGF		4 pg/ml				
	MMP-9		8.6ug/ml				
	IL-1β		138 pg/ml				
	H37Rv	Electrochemical	0.7 fM	Autolab 30	40 min	15 μl	51
	Pathogen	Multiplex PCR	–	LiquiChip200 workstation	30 min	120 μl	50
	Actotransferrin	NanoLC-MS/MS	–	Ion-trap mass spectrometer	–	200 μl	55
	High-mobility group protein B1						
	alpha 1-antichymotrypsin						
	ofilin-1						
	Neutrophil	Chemotaxis	–	Microscope	15–20 min	–	57
	Neutrophil	Chemotaxis	–	Microscope	5 min	3 μl	56
	Neutrophil	Chemotaxis	–	Modified smartphone	–	40 μl	58
Diabetes	Glucose	Colorimetric immunoassay	–	HP Scanjet 6300c	20 min	3 μl	69
	Protein						
	Glucose	Silica nanoparticle enhanced colorimetric enzymatic reaction	0.50 mM	Flatbed scanner	30 min	10 μl	64
	Glucose	Graphene and gold nanoparticles enhanced electrochemical detection	1.44 mg/dl in the interstitial fluid	Ammeter	Continuous monitoring	–	70
	HbA1c	Top-down proteomics	–	Mass spectrometer	>2.5 h	5 μl	73
	Glycated HSA						
	apoA-I						
	HbA1c	Microfluidic CE-MS	–	Mass spectrometer	–	10 μl	72
	Glycated HSA						
	HbA1c	Aptamer-based sandwich assay	–	Photomultiplier tube	30 min	100 μl	74
	Total hemoglobin						
	Serine	Enzymatic reactions	2 μM	Thermal lens detector	30 min	250	78
	Erythrocyte fragility	Osmotic lysis kinetics	–	A CCD camera and a microscope	–	–	83
	RBC deformability	Viscoelastic particle focusing	–	Optical microscope	–	–	84
	RBC deformability	Equilibrium velocity of a cell in a microchannel	–	Optical microscope	–	–	85
CKD	Blood creatinine	Electrophoretic separation, conductivity detection	87 μM	Multi-reader	Several min	–	89
	Urinary albumin	Fluorescence	5–10 μg/ml	Modified smartphone	~5 min	25 μl	91
	Urinary creatinine	Jaffe reaction, optical density	–	Photodiode	~15 min	130 μl	93
	Urinary creatinine	Enzymatic reaction, calorimetric detection	–	Quartz crystal resonator	–	100 μl	94
	Urinary creatinine	Chemiluminescence	0.07 μM	Photodiode	1 min	–	95
	Urinary protein	Colorimetric assays	8.5 μg/ml	Modified smartphone	~25 min	15 μl	92
	Cystatin C	Nonlinear threshold chemistry	–	–	4.5 h	–	90
	CRP	Colorimetric immunoassay	54 ng/ml	Modified smartphone	15 min	20 μl	96

## LoC systems for CRD diagnosis

**Fig. 2:**
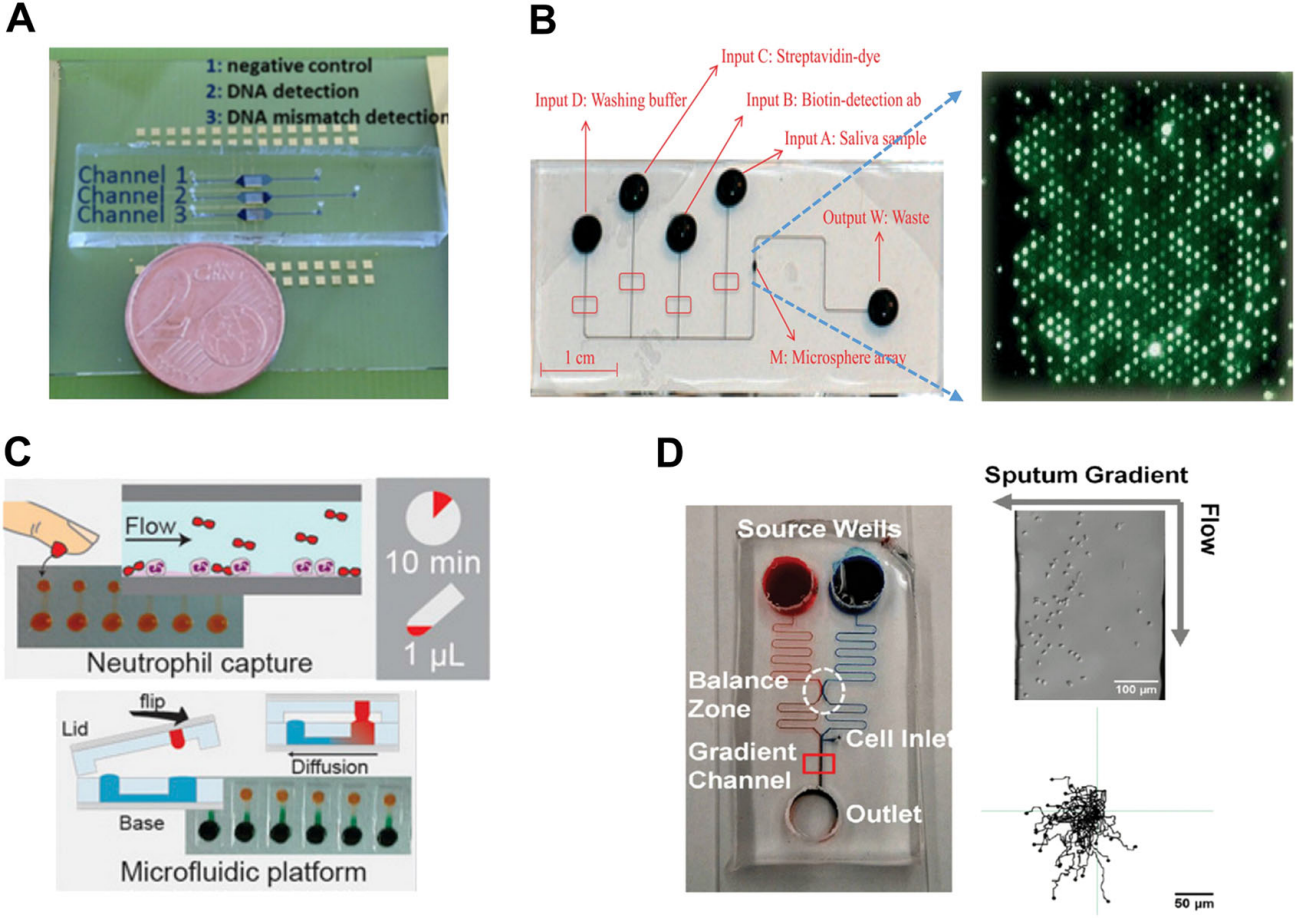
Examples of LoC-based diagnostic applications for chronic respiratory diseases. **a** A label-free microfluidic electrochemical sensor based on carbon nanotubes/ferrocene for DNA detection of *Mycobacterium tuberculosis*. The device integrates three channels for negative control, DNA detection, and mismatch DNA detection;^51^ **b** An automated and integrated LoC platform for detection of multiple CRD protein biomarkers in human saliva. Left: a photograph demonstration of the chip; Right: signal image of the microsphere array;^54^ **c** A microfluidic chip for studying the chemotactic function of neutrophils from asthma patients. The top image shows neutrophil capture from one drop of blood. The bottom image shows the diffusion-based gradient generation in a microfluidic device;^56^ **d** A microfluidic platform for evaluating neutrophil chemotaxis to the sputum samples from COPD patients. The left image illustrates the flow-based gradient generation in a PDMS device. The right image shows neutrophil migration to a sputum gradient.^57^ The figures are adapted from refs. ^51,54,56,57^ with permission from AIP Publishing for **a**, Royal Society of Chemistry for **b**, National Academy of Sciences for **c** and PLOS for **d**, respectively

According to the WHO, 235 million people have asthma; 64 million people have COPD (which is an important type of CRD); millions of people have allergic rhinitis; and many are diagnosed as having other chronic respiratory conditions.^40^ CRD cause four million deaths annually;^40^ and the burden of CRD has major adverse effects on the patients’ health and life quality. CRD are often caused by tobacco smoke, air pollution, occupational dusts and chemicals.^41^ The gold standard diagnostic methods for main CRD such as COPD and asthma include spirometry, the medical history and other clinical symptoms.^42,43^ However, the spirometry test has limited accuracy. For instance, one previous study reported that the sensitivity for diagnosing airway obstruction in COPD was 92%; specificity was 84%.^44^ The sensitivity for diagnosing airway obstruction in asthma was 29%; specificity was 90%. It is also impossible to distinguish asthma from COPD only using spirometry. Improving the diagnosis performance is important as patients with asthma need to be treated preferably with inhaled steroids. The patients also need to travel to the hospital’s laboratory to perform this test, resulting in increased cost and turn-around time of the diagnosis. Additional biomarkers can potentially improve the accuracy and timing of CRD diagnosis. In this direction, biofluids such as serum, bronchoalveolar lavage fluid, sputum, and urine are targeted for new CRD biomarkers test;^45–49^ and LoC provides a new test platform. In this section, we review some representative LoC-based methods for CRD diagnosis with the focus on different genetic and protein biomarker measurements and new cell functional assays (Fig. 2; Table 1).

Nucleic acid is an important type of biomarker for CRD diagnosis, especially for pathogen induced CRD. For instance, identifying certain nucleic acid can help diagnosing pneumonia caused by certain pathogen in COPD patients with acute exacerbation. However, the low concentration of nucleic acid in the clinical samples makes direct detection difficult without signal amplification. Ritzi–Lehnert et al. presented an automated LoC system to detect respiratory viruses from nasopharyngeal specimens.^50^ The developed disposable microfluidic processing cartridge includes a base chip, a lysis chamber with a cap and four turning valves. Liquid motion was actuated by the four turning valves. Efficient mixing was achieved by magnetic stir bars, which were integrated into the chip at the extraction, lysis, PCR and hybridization chambers. The device extracts total nucleic acids from swab samples using magnetic silica beads. The total PCR time is about half an hour while the conventional PCR takes at least several hours. Although the shorter turn-around time for CD diagnosis is not as critical as the acute infectious diseases, the faster pathogen-specific diagnosis will allow better care. However, this device still requires a benchtop operating apparatus to run the assay steps. Zribi et al. developed a label-free LoC electrochemical sensor based on carbon nanotubes/ferrocene for DNA measurement of *Mycobacterium tuberculosis* from clinical samples (Fig. 2a).^51^ Application of a fast flow generates a thin depletion layer at the sensor surface, which enhances the DNA capture rate. Thus, the LOD was enhanced from picomolar (pM) to femtomolar (fM) with a dynamic range of 0.1 fM–1 pM, while the dynamic range measured using conventional macroelectrodes is 1pM–100 nM. This is important as the pathogenic analytes are known to vary over a wide dynamic range in clinical samples. The direct genomic pathogenic identification approach without amplification step lowers the system complexity and offers an accurate pathogenic quantification method. Further integration of sample processing module that allows raw sample analysis will make it more attractive for PoC test.

Saliva is considered a diagnostic substitute of blood for protein biomarker test because it can be collected in a noninvasive manner.^52,53^ Nie et al. reported an automated and integrated LoC platform for CRD diagnosis using human saliva (Fig. 2b).^54^ Using only 10 μl of saliva, this platform can measure multiple protein biomarkers simultaneously via fluorescence sandwich immunoassays in 70 min. The reduced sample volume lowers the cost of the reagents and shortens the test time comparing to the conventional ELISA test. The multiplex detection was based on fluorescently coded microbeads, which were embedded in a microfluidic well array. The chip was used together with a customized reader device, where the automated fluidic control and optical signal detection were performed. Six inflammatory protein biomarkers in the saliva samples from patients with cystic fibrosis, asthmatic patients, and healthy subjects were compared, including human vascular endothelial growth factor (VEGF), interferon gamma-induced protein 10 (IP-10), interleukin-8 (IL-8), epidermal growth factor (EGF), matrix metalloproteinase 9 (MMP-9), and interleukin-1 beta (IL-1β). Different levels of these protein biomarkers were found between patients and healthy controls. Particularly, IP-10 showed the largest difference, suggesting IP-10 as a potential highly specific biomarker for cystic fibrosis and asthma. Furthermore, identification of IP-10 can help to determine asthma in dyspneic patients with underlying emphysematous lung. Epithelial lining fluid (ELF) contains high concentration of biological molecules in epithelial layer that directly contact cigarette smoke, making ELF another attractive target to study the physiological and pathological processes in the lung. Franciosi et al. used a commercial microfluidics-based mass spectrometer to quantitatively identify proteins in ELF from COPD patients and healthy controls.^55^ The results revealed the different levels of lactotransferrin, high-mobility group protein B1, alpha 1-antichymotrypsin and cofilin-1 in ELF from COPD patients and the controls.

CRD have long been associated with inflammatory cell recruitment to the airway. Therefore, the inflammatory cell trafficking has been proposed as a potential biomarker for CRD. Sackmann et al. developed a microfluidic chip to study the chemotactic function of neutrophils from patients with asthma (Fig. 2c).^56^ An attractive feature of this device is the on-chip neutrophil isolation directly from a drop of whole blood, which was achieved by coating a layer of specific cell adhesion molecule in the channel. The chemoattractant gradient in the microchannels is created by placing a lid with chemoattractant solution to the base of the device. The cell migration speed in the chemical gradient was measured. The results showed that the chemotaxis ability of neutrophils is significantly lower from asthmatic patients compared with healthy controls. Similarly, we developed a microfluidic platform for studying neutrophil chemotaxis to the sputum from patients with COPD (Fig. 2d).^57^ The device used a passive gravity pumping method to generate a sputum supernatant gradient for neutrophil migration experiments. Neutrophils from third party healthy individuals were used as the responding cells to compare the chemotactic ability of the sputum from COPD patients and healthy controls. Our results show the elevated neutrophil chemotaxis to the sputum from COPD patients, which was correlated with the spirometry data. More recently, we developed a smartphone-based system to rapidly provide digitalized test results for neutrophil chemotaxis in a microfluidic device.^58^ This system was validated by testing neutrophil chemotaxis to the sputum from COPD patients and on-site tests in the hospital, suggesting the potential of implementing digital and mobile technologies for cell functional-based disease diagnosis.

In summary, LoC-based platforms make the genetic and protein CRD biomarkers detection more easily and quickly. It also enables high-throughput detection of a panel of different CRD biomarkers, providing useful diagnostic information for making medical decision. The rapid assessment of cell chemotaxis using the microfluidic devices offers a promising new method for CRD diagnosis. However, these new potential molecular or cell-based biomarkers require extensive further research and clinical validations to develop practical applications.

## LoC systems for diabetes diagnosis

**Fig. 3:**
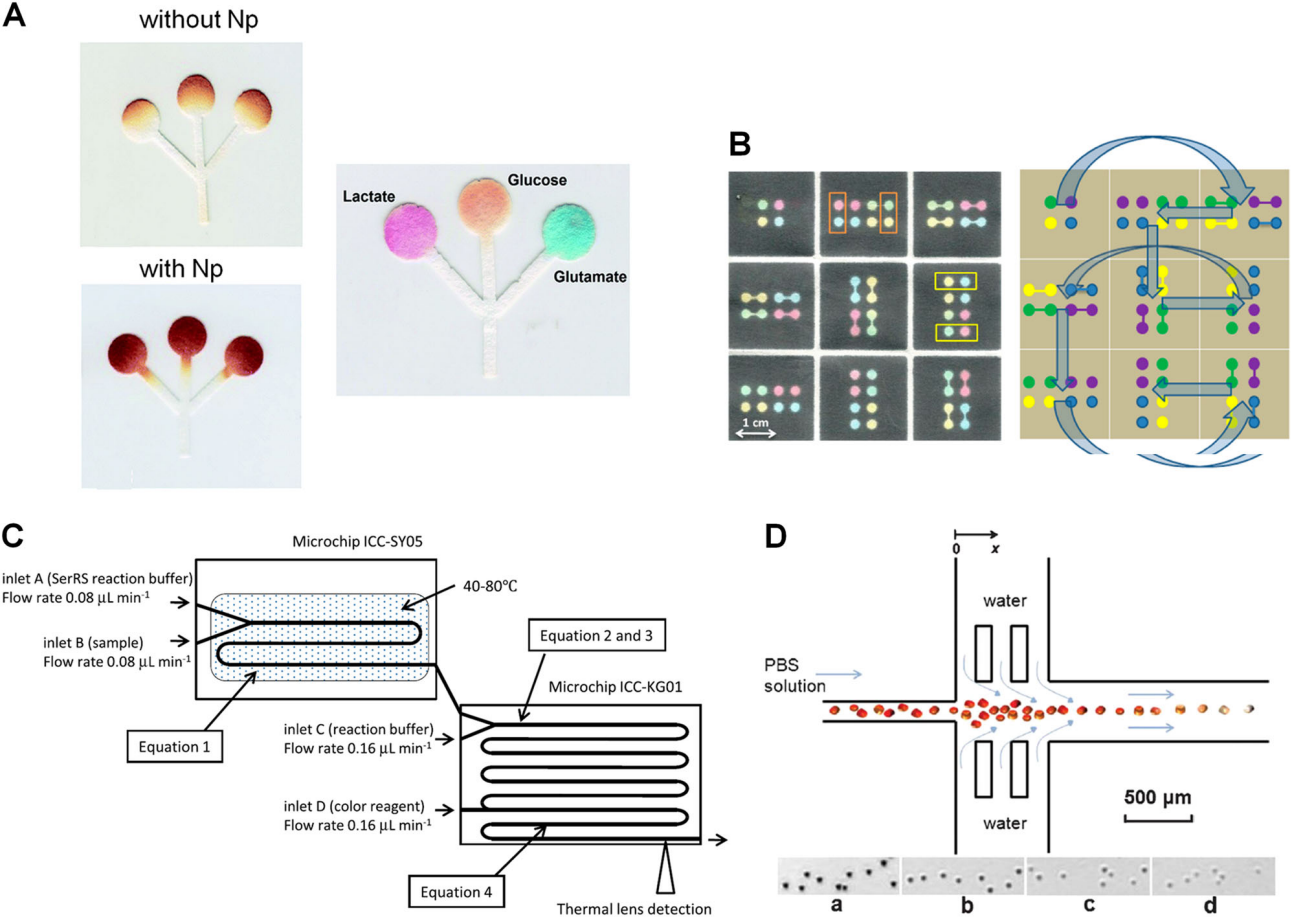
Examples of LoC-based diagnostic applications for diabetes. **a** (Left) Illustration of a µPAD to test glucose with (left bottom) and without (left top) SiO_2_ nanoparticles modification; (Right) Illustration of the proposed μPAD showing the analysis of an artificial urine sample spiked with lactate, glucose, and glutamate;^64^ **b** An easy and low-cost three-dimensional microfluidic paper device for glucose assay. The right image shows the flow pattern of the device;^69^ **c** A microfluidic biosensor for the detection of amino acids using enzymatic reactions coupled with spectrophotometric detection;^78^ **d** A simple microfluidic tool for erythrocyte fragility study by analyzing osmotic lysis kinetics under hydrodynamic focusing. The inset images (**a**–**d**) show the cell images at various locations.^83^ The figures are adapted from refs. ^64,69,78,83^ with permission from Royal Society of Chemistry for **a**, **d**, American Chemical Society for **b**, and Springer for **c**, respectively

Diabetes or diabetes mellitus (DM) is a metabolic disease in association with high blood sugar level over a prolonged period.^59^ The number of people with diabetes was 422 million in 2014;^59^ and the global prevalence of diabetes among adults over 18 years old was 8.5% in 2014. The existing therapeutic strategies for treating diabetes are only partially successful. Therefore, diabetes screening and early identification of the patients at high risk is important for reducing diabetes-associated complications. For example, fast blood glucose, which measures blood glucose after fasting for at least 8 h, is the most common marker used for clinical DM screening and diagnosis. Many blood glucose meters are already available for self-monitoring of the blood glucose level. Most of the test strips in the market are still expensive (typically cost 1–2 US dollars), which is unaffordable in many developing countries for routine blood glucose test. Furthermore, the accuracy of existing glucose meters remains low. The US Food and Drug Administration (FDA) has set the accuracy criteria for the glucose meters to be ±20 mg/dl for the glucose level <100 mg/dl or ±20% for the glucose level >100 mg/dl for at least 95% of results. However, significant variations in accuracy exist among different and within each meter brands. The FDA reported 100 deaths associated with potential glucose meter inaccuracies between 1992 and 2009; and 12,672 serious associated injuries from 2004 to 2008.^60^ In one study, researchers used Monte Carlo simulation to evaluate the clinical significance of glucose meter precision.^61^ The results demonstrated that the variability of 10% in a glucose measurement can result in change of insulin dosage of 16–45%, and the dosage change could be two-fold or even greater with the glucose measurement variability of >10–15%. The study suggested that the total precision of <1–2% of a glucose meter was necessary to obtain comparable insulin dosage using the laboratory analyzers. However, most of the commercial glucose meters cannot meet this precision requirement. In this direction, LoC technology has the potential to further improve the PoC glucose test at lower cost and higher accuracy. In this section, we review some representative LoC-based methods for DM diagnosis, which target glucose, different protein and genetic biomarkers, as well as red blood cell (RBC) functions (Fig. 3; Table 1).

Many microfluidic paper-based analytical devices (µPADs) have been developed for glucose detection. Compared with the conventional glucose test strips, µPADs introduce hydrophobic barriers in hydrophilic paper to confine the fluid flow within a desired location, which improves the efficiency and control of the reagents and allows measuring multiple analytes simultaneously. In addition to some common methods for µPAD fabrication such as wax printing, photolithography and screen-printing, other simpler approaches were developed. For example, Garcia et al. used a handheld metal stamp to fabricate the device.^62^ Briefly, a native paper was covered by a paraffinized paper. The pattern in the metal stamp was transferred to the native paper by heating the metal stamp and pressing it against the paraffinized paper. The final cost of one µPAD was only ¢4. Besides glucose, the detection of uric acid, bovine serum albumin, and nitrite were successfully performed in their device. In addition, covalent coupling of enzymes on the paper surface by chemical modification improves the color uniformity inside the sensing area, which significantly decreases the test variation. The semi-quantitative analysis of all four analytes in artificial urine samples revealed an error smaller than 4%. Oyola–Reynoso et al. used a ball-point pen to draw the hydrophobic regions using silane/hexane ink.^63^ Researchers also reported the integration of novel nanomaterials with µPAD to enhance the detection signal. Evans et al. added silica nanoparticles to the device that improves the intensity and uniformity of the color signal (Fig. 3a).^64^ The LOD of glucose using this method was 0.5 mM. Palazzo et al. coupled gold nanoparticles with µPAD for colorimetric glucose detection. This method avoids color bleaching, which would happen in the conventional bioenzymatic devices.^65^ Beside the colorimetric detection method, many µPAD used the electrochemical method to detect glucose at high sensitivity, either by integrating the commercial screen-printed electrodes with the μPAD or directly printing the electrodes on paper substrates. Because signal detection of µPAD-based methods is highly compatible with the conventional glucose meters, current signal readers can be easily adapted. More details about µPAD-based glucose detection is provided in a recent review article.^66^ Although µPAD showed great potential, further research on better understanding capillary wicking to provide more precise flow control and finding methods to prevent evaporation on the paper sheets will be necessary to improve the detection accuracy. Compared with blood test, urine tests are not commonly done for diabetes diagnosis. On the other hand, urine glucose measurement is useful for DM screening and the urine protein level is an indicator of kidney damage.^67,68^ In order to provide an easy and low-cost routine test, Sechi et al. developed a three-dimensional (3D) microfluidic paper device, which can be used to analyze urine, blood, and saliva (Fig. 3b).^69^ The fabrication of this paper device is based on wax printing technique and the basic principle of origami. Using this method, urinalysis of protein and glucose assays were realized, suggesting its potential to enable routine DM monitoring. The continuous glucose monitoring is very important for the treatment of diabetes because glucose controls the formation of advanced glycation end products, which leads to reduction of the HbA1c level. Currently, the implantable enzyme electrode sensors are frequently used for continuous glucose monitoring in clinics. However, those sensors are limited by their invasive measurement, short lifetime of the electrodes, and the interference from body bioelectricity. Interstitial fluid (ISF) has been proposed to be an alternative non-invasive sample for continuous glucose monitoring because the glucose concentrations in the ISF are closely related to those in the blood. Pu et al. developed a PDMS microfluidic chip to non-invasively extract the ISF from the subcutaneous tissue.^70^ A graphene-modified electrochemical sensor was integrated into the chip to enable continuous detection of glucose. The single-layer graphene gold nanoparticle coating on the working electrode improves the resolution of the glucose measurements. This sensor could precisely measure glucose in the linear range from 0 to 162 mg/dl with a LOD of 1.44 mg/dl; and it has the potential to address the current clinical challenge of continuous glucose monitoring for hypoglycemia diagnosis. The flexible PDMS material also made this sensor wearable.

Another widely accepted marker for DM diagnosis is glycated hemoglobin A1c (HbA1c).^71^ HbA1c refers to the average hemoglobin (Hb) glycation level inside the RBCs for the preceding 2−3 months, which is more stable compared with fluctuation of the blood glucose level. Clinical measurement of HbA1c is typically based on separation methods or immunoassays, such as high-performance liquid chromatography (HPLC) and immunoturbidimetry. In addition, MS was used as a potential method for assessing Hb glycation. However, these methods are expensive and complicated. LoC technologies offered miniaturized DM biomarker detection platforms, such as different LoC-based MS systems. Erin et al. developed a microfluidic CE-MS to assess HbA1c in whole blood lysate.^72^ Different sub-units of α-Hb, β-Hb and glycated β-Hb could be resolved using this method. They found the glycated β-Hb correlated well with the HbA1c levels derived in the clinic. In addition, glycation of human serum albumin (HSA) can also be measured using this technique. The simultaneous detection of Hb and HSA glycation shows the capacity to provide more complete information for DM control. Mao et al. developed a silicon nanoLC-MS to measure glucose, HbA1c, glycated HSA, and glycated apolipoprotein AI at the same time using only 5 µL of blood.^73^ A multi-nozzle emitter array chip, which consists of a trap column and a nanoLC column in a three-layer Si–Si–glass structure, was used to enable on-chip sample separation. Using this system, the levels of multiple protein DM biomarkers were quantified; and the results can separate healthy controls from Type 2 diabetes patients. Although the LoC-based MS systems can detect multiple markers and resolve different protein isoforms, it requires further development to simplify device fabrication and test operation. Using an aptamer-based approach, Li et al. developed a microfluidic system for automatic and simultaneous measurements of HbA1c and Hb.^74^ Specific capture aptamers were coated on magnetic beads and mixed with whole blood. The mixture was loaded to a microfluidic chip and an external magnetic field was used to collect the target–aptamer–bead complexes during the washing step. The signal was detected after adding second fluorescence-labeled aptamers. Compared with the traditional bench top HPLC system, this system reduced the reagent consumption by 75% and decreased the analysis time from 3.5 h to 30 min.

The amino acid content is also useful for the diagnosis of diabetes.^75,76^ In one study, five-branched chain and aromatic amino acids were reported to be significantly associated with future diabetes, including isoleucine, leucine, valine, tyrosine, and phenylalanine.^77^ A combination of three amino acids could predict future diabetes. But the conventional detection methods for amino acid such as HPLC and liquid chromatography-MS (LC-MS) require cumbersome equipment and the techniques are time-consuming and costly. Kugimiya et al. developed a microfluidic biosensor for the detection of amino acids using enzymatic reactions and spectrophotometric detection (Fig. 3c).^78^ This system contains two serially connected identical chips. Each of them has two chemical inlets and a serpentine channel for reaction. This LoC-based reaction system has the advantages in reduced analyte consumption and rapid reaction. To detect serine, the seryl-tRNA synthetase was used for hydrogen peroxide production, which was measured using the Trinder reagent spectrophotometric method. Although serine has not been reported to be directly associated with diabetes, the developed LoC detection system has the potential to be applied for the detection of the diabetes-associated amino acids, and thus it can assist early diabetes risk assessment.

In addition to the molecular biomarkers, cell deformability has been considered as a new biomarker for DM diagnosis.^79–81^ For example, erythrocyte (RBC) fragility can provide critical information of DM. In DM, the change of the ratio between the phospholipids and cholesterol results in a decrease in the erythrocyte deformability.^82^ Zhan et al. used a simple microfluidic tool to study erythrocyte fragility by analyzing osmotic lysis kinetics (Fig. 3d).^83^ Two water flows from the two side inlets hydrodynamically focus erythrocytes injected from the central inlet. The flow rate of the cell sample and water can vary to obtain different focusing effects. The lysis kinetics was traced by measuring the release of intracellular contents. The change of RBC fragility induced by glucose incubation was detected using this platform at high sensitivity. In another study, Cha et al. developed a method for measuring cell deformability based on the 3D viscoelastic particle focusing.^84^ Using this technique, cells were efficiently delivered at the stagnation point of the cross-slot channel; and the cell stretching under flow was monitored. The deformability change of non-spherical RBC was characterized using this method. Furthermore, this method showed decreased deformability of human mesenchymal stem cells caused by nutrient starvation. The transit time of a cell through a microchannel can also be used to evaluate cell deformability. Tsai et al. proposed a new “equilibrium velocity” of cells in the microfluidic channel, which is directly related to cell stiffness.^85^ Based on this concept, the stiffness of RBC from a healthy subject and a diabetes patient was evaluated using a microfluidic device and the results showed that the deformability of the patient’s RBC was lower than the healthy subject. Thus, these studies suggest the potential of cell deformability measurement for detecting pathological changes in diabetes.

Collectively, LoC-based systems offered new approaches for DM diagnosis, which target a broad range of DM biomarkers including glucose, proteins, amino acids and cell properties.

## LoC systems for CKD diagnosis

**Fig. 4:**
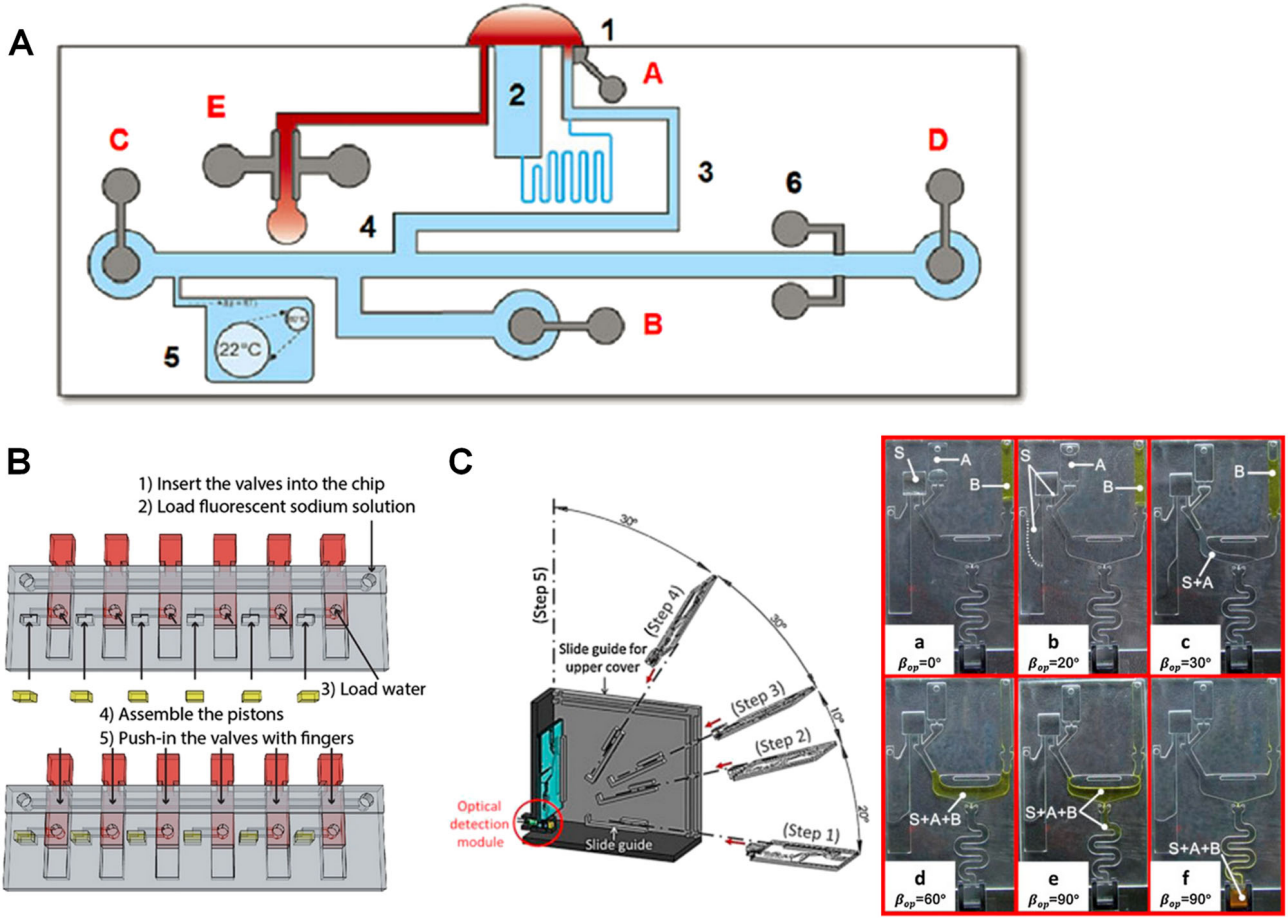
Examples of LoC-based diagnostic applications for chronic kidney disease. **a** A PoC device to measure the elevated creatinine level in blood based on electrophoretic separation and conductivity detection. The different functional units include: [1] sample opening; [2] evaporation reservoir; [3] Cations injection channel by moving boundary electrophoresis; [4] double-T injector; [5] reservoir with gas bubble for liquid expansion control; [6] conductivity detection electrodes; [A, B] high-voltage injection anode and cathode; [C, D] high-voltage separation anode and cathode; [E] electrodes to measure sample conductivity;^89^ **b** A microfluidic device integrated with 3D printed movable components and a smartphone imaging platform for colorimetric urinary protein quantification;^92^ **c** A microfluidic chip integrated with the capillary–gravitational valves for urinary creatinine measurement. Left: Scheme of the rectangular hand-assist kit and its operation processes. Right: The sequential functions of chip for creatinine assay as the angle of the chip was changed;^93^ The figures are adapted from refs. ^89,92,93^ from Royal Society of Chemistry for **a**, American Chemical Society for **b**, and Elsevier for **c**, respectively

Patients with CKD suffer from gradual loss of kidney functions over time. CKD affects 8–16% of population worldwide^86^, and the main causes of CKD are diabetes and high blood pressure.^86^ The most common two markers for renal function assessment are the estimated GFR (eGFR) and the urine albumin to creatinine ratio (UACR). eGFR is usually calculated based on the creatinine level in the blood. Recently, cystatin C in the blood has been reported to be a more stable marker to estimate GFR. In this section, we review some representative developments of LoC detection systems for both established and newly proposed CKD biomarkers (Fig. 4; Table 1).

Creatinine is a waste product that forms when creatine breaks down. Creatinine is one of the most widely used markers of renal function, and the blood creatinine level can be used to calculate eGFR.^87^ Clinical detection of creatinine concentration uses benchtop chemical analyzers based on either the Jaffe reaction or enzymatic reaction. But these types of equipment are expensive and usually only available in centralized labs. PoC devices based on the similar detection principles are also available to measure blood or serum/plasma creatinine.^88^ For instance, the Nova StatSensor Creatinine meter is an electrochemical handheld analyzer and miniaturized, disposable biosensor for whole blood creatinine test. It can give out the result in 30 s using 1.2 µL of whole blood with a measurement range of 27–1056 μM. However, the current PoC devices are still expensive and show poor detection precision at low creatinine concentrations.^88^ LoC-based devices have the potential to provide more cost-efficient and accurate test for creatinine. Ávila et al. developed a LoC device to measure the elevated creatinine level in blood, which is based on electrophoretic separation and conductivity detection (Fig. 4a).^89^ The whole test can be done in an automated manner using a single disposable microfluidic chip and a handheld analyzer. Although this method has a higher LOD of ~100 μM, it can still be used as a screening tool for distinguishing renal insufficiency patient (>100 μM in serum) and healthy subject (60–100 μM in serum). As mentioned before, cystatin C in the blood has been proposed as another marker for estimating GFR. Cystatin C is a 13 kDa protein that is filtered by the glomerulus and reabsorbed and metabolized by tubular cells. Compared with creatinine, cystatin C production is more stable and not dependent on muscle mass. Huynh et al. combined threshold chemistry and microfluidics to allow digital colorimetric detection with naked eyes for cystatin C concentration in serum up to 1.5-fold increase, which can indicate the progression from normal kidney function to stage 3 CKD.^90^ The main advantage of the threshold chemistry is that it can detect a small increase in the target protein concentration that matches the sensitivity requirement of the assay set by the tunable threshold concentration.

Compared with blood, urinary sample can be obtained noninvasively. Therefore, urine biomarkers are more suitable for PoC test. For example, UACR is a clinical marker for routine screening of renal function. A calculated UACR of 30 mg/g or higher is an indication of renal damage. Increasing number of studies developed LoC devices for the measurement of creatinine and albumin in urine samples. Coskun et al. developed a fluorescence-based urinary albumin test, which only requires 148 g of sample and can be attached to the camera of a smartphone for reading.^91^ The LOD of this test is 5–10 μg/mL, which is three times lower than the normal range. It was envisioned that this system can be used for early diagnosis and monitoring of CKD, diabetes, and hypertension. Chan et al. used a 3D printer to fabricate a series of movable microfluidic chip components, including torque actuated pump and valve, rotary valve and pushing valve (Fig. 4b).^92^ These components can be operated manually without external pressure sources and are easy to manipulate. They were then integrated into a microfluidic device for colorimetric urinary protein quantification. A smartphone was also used as the imaging platform. Wang et al. developed a capillary–gravitational valves in a plastic chip for urinary creatinine measurement.^93^ This chip used the balance between capillary and gravitation forces for sequential fluid delivery, metering and mixing, which can be controlled by battery powered motor or simply operated by hand in resource-limited settings (Fig. 4c). With proper channel structure design, the burst pressure of the capillary values could be broken by the gravitational force while the chip was rotated from horizontal to vertical. They verified the performance of the designed chip to be consistent with the clinical method using real urine samples. David et al. developed a calorimetric sensing microchip to measure creatinine in urine.^94^ An array of microfabricated Y-cut quartz resonators was integrated into the sensor. Urinary creatinine was transduced into temperature signatures by alginate-entrapped creatinine deiminase, which permits the quantification of creatinine. The results showed good agreement in five urine samples using calorimetric and HPLC methods. One major limitation of the existing creatinine biosensors was the interference of other analytes in the biological fluids, such as serum and urine, resulting in inaccurate and biased detection. The LoC-based sensors have the potential to overcome this issue and provide more accurate detection. For example, Hanif et al. reported that creatinine can generate chemiluminescence when reacting with hydrogen peroxide and the reaction is remarkably enhanced in the presence of cobalt ions.^95^ Using this phenomenon and a flow mixing microfluidic device, they developed a chemiluminescence creatinine sensor with a LOD of 0.07 µM and linear range of 0.1–30 µM, which is much more sensitive than most reported methods and the commercial PoC sensors. They validated this method by detection of creatinine in human urine samples and the test took less than 1 min. The recovery of creatinine in spiked urine samples was ~100% and the interference of some common species in urine, such as amino acids, ascorbic acid, and creatine, is negligible. However, at this stage, none of these LoC-based methods can simultaneously measure albumin and creatinine to predict UACR.

In addition to these traditional CKD biomarkers, researchers also explored the use of LoC technologies for detection of new biomarkers, such as CRP. For example, we recently reported a paper-based microfluidic chip for rapid and low-cost detection of CRP level in CKD patients.^96^ The colorimetric detection signal of the gold nanoparticle-based immunoassay can be analyzed easily and digitally by a smartphone. Our results showed the expected higher CRP level in CKD patient’s serum and plasma compared with healthy subjects.

In summary, LoC technologies enabled miniaturized test platform for detection of different biomarkers in blood and urine to assess the renal function. Both the 3D printed movable chip and the capillary and gravity-based fluid transport strategy eliminate the use of an external instrument for the fluid handling. They allow easy manual operation by the end user, and the color-based test signal can be digitally read by a smartphone. These useful features suggest the potential of the LoC-based methods for future self-assessment of kidney function by the patients at home. The current 3D printing technology has the limitation for mass production of the LoC diagnostic device. In this regard, once the prototype device is validated, the device fabrication can be switched to the ones more suitable for mass production, such as injection mold.

## Concluding remarks

This review summarizes the recent development of LoC-based technologies for CD diagnosis with the focus on CRD, diabetes and CKD. Other CD such as cancers are not included in this review. Owing to miniaturization, these new LoC-based systems showed promise to improve test speed, throughput and cost efficiency.

Looking forward, this new research field is facing a number of challenges while the efforts to address these challenges present exciting opportunities. For example, most current LoC systems need to demonstrate their competitive advantages over conventional systems in cost efficiency and test accuracy. For CRD, the spirometry is still the gold standard diagnosis method and many molecular biomarkers are still under investigation. The LoC systems do not replace the spirometry but they can play important roles in identifying and validating new biomarkers. For diabetes, the µPAD has shown great potential to significantly reduce the cost compared with the existing glucose meters, and thus it can benefit the patients with diabetes particularly in developing countries. Further development of LoC-based wearable system for continuous glucose monitoring may provide an effective tool for the glucose control in patients with type 2 diabetes. For CKD, the urinary biomarkers such as UACR are preferable for PoC test as these tests are non-invasive but the test accuracy is often interfered by other analytes in urine. The LoC-based sensors have the potential to address this issue with their better ability for sample processing and analyte enrichment. New reactions such as enhanced chemiluminescence reaction of hydrogen peroxide with creatinine can improve the detection accuracy as well as reduce the cost for the analyzer by using a simple photodiode. In general, the LoC-based methods are not necessarily to replace the conventional methods but can be useful alternatives or complementary tests with their unique features to the conventional methods.

In addition, the label-free detection and multi-biomarker analysis are both attractive approaches to improve CD diagnosis. On the other hand, they impose competing technical requirements in terms of simplicity versus specificity and throughput. This aspect is also related to the challenges to integrate multiple diagnostic tests in a single platform for different types of biomarkers such as DNA, proteins and cells, and different samples such as blood, urine, sputum and saliva. The facts that many CD are closely associated and often share common biomarkers suggest the opportunity to develop integrated diagnosis methods for different CD. Research and development in this direction requires innovative approaches and design optimization. LoC-based CE-MS or LC-MS systems is a promising new approach owing to the unique ability of these detection methods to separate analytes of extremely low concentration with high efficiency at high speed.

Furthermore, clinical acceptance of new diagnostic technology not only requires improved and reliable test performance but also demand significant new benefits in terms of test operation, data analysis, result reporting and management. However, few current LoC systems can meet all these requirements. Many assays still require the use of additional equipment for sample preparation and signal detection and analysis. The highly integrated, low-cost and automated LoC systems combined with portable device for test signal reading, analysis and communication are required. In this context, recent fast development of MS^2^ technology (mobile sensing based on microfluidic devices and smartphone) offers the most promising approach for rapid PoC CD diagnosis.^97^ On the chip development side, new approaches and methods for rapid prototyping and large-scale manufacturing of microfluidic devices with improved functionality are expected to rapidly grow and make major advancement in the near future. Finally, as seen from some examples reviewed in this article, cell-based tests provided invaluable information for CD assessment. Targeting altered cell functions in CD presents a novel approach for disease diagnosis and assessment at a higher biological level. On the other hand, cell functional tests have much higher technical requirement to allow more complicated assays. Therefore, it has been a major challenge to establish the cell functional assays for disease diagnosis in clinical practice. In this regard, we envision that integrated LoC systems will provide a promising approach for cell functional assays to facilitate CD diagnosis in the future.
